# MicroRNA: Another Pharmacological Avenue for Colorectal Cancer?

**DOI:** 10.3389/fcell.2020.00812

**Published:** 2020-09-02

**Authors:** Xueliang Wu, Fuguo Yan, Likun Wang, Guangyuan Sun, Jinyu Liu, Ming Qu, Yicheng Wang, Tian Li

**Affiliations:** ^1^Department of General Surgery, First Affiliated Hospital of Hebei North University, Zhangjiakou, China; ^2^Department of General Surgery, Xinchang Hospital of Wenzhou Medical University, Xinchang, China; ^3^School of Basic Medicine, Fourth Military Medical University, Xi’an, China

**Keywords:** microRNA, colorectal cancer, carcinogenesis, apoptosis, therapeutic target

## Abstract

MicroRNAs (miR) are single-stranded RNA of 21-23 nucleotides in length that repress mRNA translation and induces mRNA degradation. miR acts as an endogenous factor of gene expression and plays a crucial part in cancer biology such as cell development, proliferation, differentiation, and apoptosis. Numerous research has indicated that dysregulation of miR associates with colorectal carcinogenesis. In this review article, we firstly introduce the background of miR and colorectal cancer, and the mechanisms of miR in colorectal cancer, such as the proliferation, apoptosis, and progression. Then, we summarize the theranostic value of miR in colorectal cancer. Eventually, we discuss the potential directions and perspectives of miR. This article serves as a guide for further studies and implicate miR as a potent theranostic target for colorectal cancer.

## Introduction

Cancer, cardio-cerebrovascular disease, and nervous system disease are major causes of mortality worldwide (Li et al., 2017, 2018, 2019). Colorectal cancer, also called as bowel cancer, is malignancy developed in the colon or rectum (Kuipers et al., 2015; Shi et al., 2018). According to the Global Cancer Statistics estimating incidence and mortality worldwide for 36 cancers in 185 countries, more than 1.8 million new colorectal cancer cases and 881,000 deaths are estimated to occur in 2018. Overall, colorectal cancer ranks third in terms of incidence and second in mortality (Bray et al., 2018). The majority of patients fails to be diagnosed until a middle/advanced stage, thus usually with a bad outcome. Furthermore, the available treatments are shown to be marginally effective with various adverse effects. Thereby, it is urgent to explore novel theranostic targets to improve the curative effects and outcomes of colorectal cancer.

MicroRNAs (miR), firstly discovered in 2001 (Lagos-Quintana et al., 2001; Lau et al., 2001), are single-stranded RNA of 21–23 nucleotides in length that can repress mRNA translation and induce mRNA degradation (Li and Rana, 2014; Mehta and Baltimore, 2016). They are a subclass of short non-coding RNA molecules in eukaryotic cells and control the expression of 60% of the human protein-coding genes, approximately (Akbari Moqadam et al., 2013). So far as the status, 38,589 hairpin precursors and 48,860 mature miR from 271 organisms have been annotated in mirBase (Kozomara et al., 2019). Generally, a miR may target multiple genes, vice versa, leading to a potentially complicated miR-mediated signaling network. Numerous research has demonstrated that miR play pivotal roles in colorectal cancer (Tian et al., 2019), hepatocellular carcinoma (Xu et al., 2019), gastric cancer (Zhang et al., 2019), lung cancer (Parayath et al., 2018), etc. Dysregulation of miR may regulate the development, proliferation, apoptosis, and progression of colorectal cancer in human beings. In light of their emerging roles, we carefully reviewed previous publications and completed this manuscript.

This review article is intended to discuss the diverse roles of miR in colorectal cancer. Because most literature focused on colon cancer has been reviewed, we will pay more attention to rectal cancer, as well as colon cancer partly from recent research literature. We firstly introduce the background of miR, cancer biology, colorectal cancer, as well as the roles of miR in colorectal carcinogenesis. Then we focus on the mechanisms of miR in colorectal cancer, including proliferation, apoptosis, progression, etc. Thirdly, theranostic value of miR in rectal cancer will be discussed, such as diagnosis, staging, therapy, and prognosis. Ultimately, we discuss the potential directions of miR. This article summarizes recent literature and provides an elaborate picture of miR.

## Mechanisms of miR in Colorectal Cancer

### Colorectal Carcinogenesis

**FIGURE 1:**
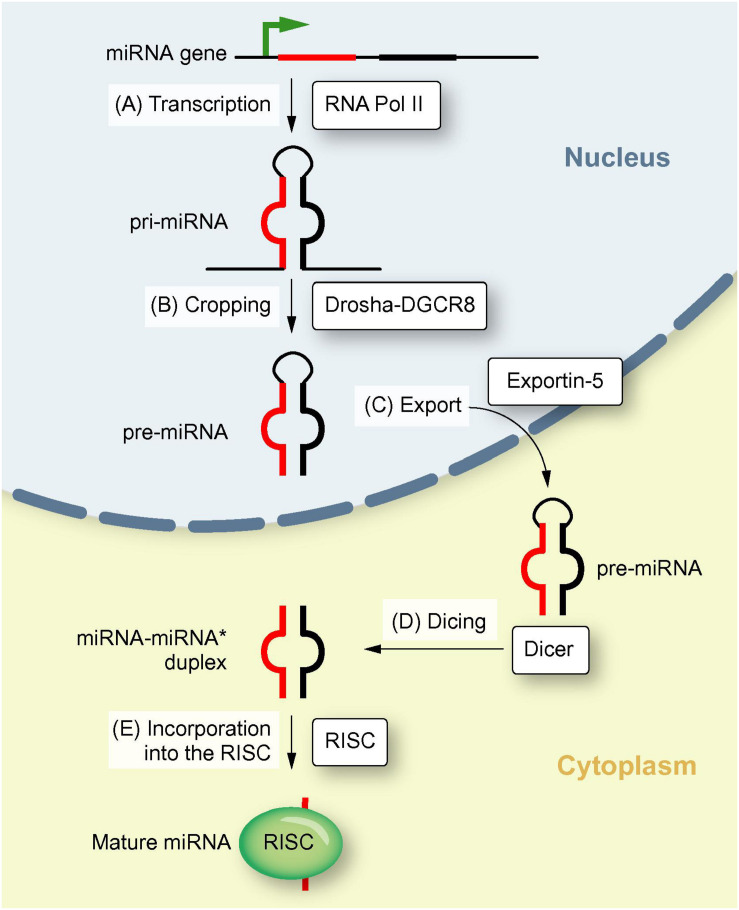
Biogenesis of miR. This illustration shows the biogenesis of miR, including transcription of a pri-miR, formation of pre-miR, translocation to the cytoplasm and maturation of the miR (Reproduced with permission) (Borel et al., 2012).

miR were firstly discovered and reported in 2001. Ambros pointed out miR are highly conserved among vertebrates, invertebrates, and plants. miR gene is localized throughout the whole genome. Biogenesis of miR is pivotal in life science (Figure 1) that is involved in a variety of human diseases. Readers can refer to these narrative review for a detailed understanding (Romero-Cordoba et al., 2014; Vishnoi and Rani, 2017).

So far as the status, the main mechanisms and processes of colorectal carcinogenesis are partly elucidated. In general, there are two major pathways of colorectal pathogenesis: the traditional adenoma–carcinoma pathway (namely the chromosomal instability sequence, accounting for 70–90% of all), and the serrated neoplasia pathway (10–20% of all). These pathways represent distinct multiple genetic and epigenetic events in a rather sequential order (Cancer Genome Atlas Network, 2012; Dekker et al., 2019). Chromosomal instability phenotypes typically develop following genomic events initiated by an APC mutation, followed by RAS activation or function loss of TP53. On the contrary, the serrated neoplasia pathway is associated with RAS and RAF mutations, and epigenetic instability, characterized by the CpG island methylation phenotype, which results in microsatellite stable and instable cancers (Dekker et al., 2019). Recent findings have demonstrated that miR (Wan et al., 2020), apoptotic pathways (Abraha and Ketema, 2016), tumor-derived exosomes (Mousavi et al., 2019), RAS signaling (Bahrami et al., 2018), colorectal cancer stem cell (Tao and Zhu, 2011), leptin (Zhou et al., 2014) may act as therapeutic targets, whereas a detailed description is beyond the scope of this review.

**FIGURE 2:**
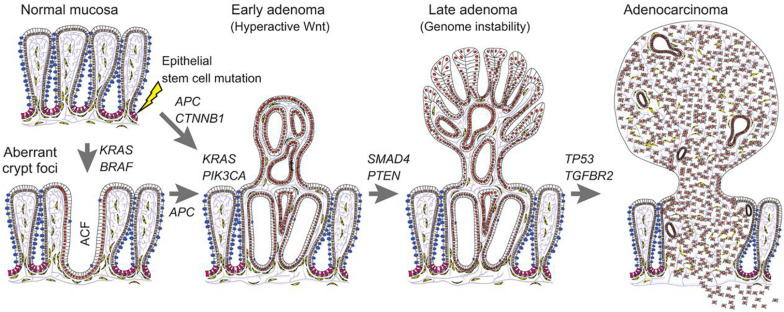
Carcinogenesis in colorectal cancer. This figure suggests the carcinogenesis process of colorectal cancer, from normal mucosa to adenocarcinoma (Reproduced with permission) (Strubberg and Madison, 2017).

Carcinogenesis is a pathological alteration characterized by abnormal epithelial-mesenchymal transition (EMT) (Wang et al., 2020), which is a process of the EMT (Jiang et al., 2018;, Shi et al., 2018) (Figure 2). So far as the status, the mechanisms of colorectal carcinogenesis remain still elusive. Recent research has demonstrated that a series of signalings/pathways are involved in the development and oncogenesis of colorectal cancer, such as Wnt (Kleeman et al., 2019), TGF-β (Calon et al., 2012), K-ras (Codacci-Pisanelli et al., 2009), PI3K (Zhang et al., 2011), and p53 (Seton-Rogers, 2015). miR can regulate some pivotal oncogenes and anticancer genes, thus, dysregulation of miR may contribute to the carcinogenesis of tumors.

Compared to para-carcinoma tissues, there is defect in miR synthesis and dysregulation in cancer tissues (Lu et al., 2005; Melo et al., 2010). Over 50% of human miRs locate at the tumor-related genomic regions (fragile sites), suggesting the potential involvement of miR in carcinogenesis (Lagos-Quintana et al., 2002). Previous research has reported the altered expression levels of specific miR in the tissues of breast cancer, lung cancer, colorectal cancer, compared to the normal (Takamizawa et al., 2004; Lin et al., 2005). Pradhan et al. (2015) presented a systematic biology approach to the understanding of the miR-regulatory network in colorectal cancer. They obtained an initial set of significant genes in colorectal cancer by mining relevant literature from three databases: miRBase, miRWalk, Targetscan, and Gene Expression Omnibus (GEO) microarray. Thereafter, they identified 3 colorectal carcinogenesis-related novel miR (hsa-miR-630, hsa-miR-100, and hsa-miR-99a) in the global miR-gene network using topological and sub-graph analyses, which are involved in the pathogenesis of colorectal cancer (Pradhan et al., 2015). Wang et al. (2012) applied stem-loop realtime polymerase chain reaction (PCR) to quantitatively detect miR-34a, miR-155, and miR-200c expression in 109 pair-matched human colorectal cancer mucosa and the corresponding normal mucosa. They found that miR-34a, miR-155, and miR-200c were all expressed at higher levels in colorectal cancer. In rectum, miR-34a and miR-200c were upregulated. Besides, miR-34a expression was higher in rectal cancer having more advanced TNM stage (node metastasis number, distant metastasis, tissue grade, etc.) (Wang et al., 2012).

### Proliferation and Apoptosis

Apoptosis is programmed cell death existing in almost all cells (Ferrari et al., 2018; Tabari et al., 2019) and miR play a pivotal role in tumor apoptosis (Agostini et al., 2010). Antisense miR-targeted survivin can inhibit proliferation, colony formation, and invasion of HRC-9698 cell, thereby, which further induces its apoptosis (Dai et al., 2015). It was found that the expression levels of miR-144 were significantly reduced in the SW837 and SW1463 cell lines, and the overexpression of miR-144 suppressed cell viability, and proliferation. In addition, Rho-associated coiled-coil containing protein kinase 1 (ROCK1) was identified as a target of miR-144 in the rectal cancer cells. Mechanically, the supplementation of ROCK1 markedly restored the cell migration and proliferation, the process of which can be inhibited by miR-144 (Cai et al., 2015).

Proliferation means rapid growth or reproduction of cell (Ferrari et al., 2018). miR-451a inhibits tumor cell proliferation and attenuates surviving fraction. Notably, inhibition of miR-451a increases tumor sphere formation, proliferation, and surviving of tumor cells. Ruhl et al. (2018) used miR expression profiling and discovered a set of miR were upregulated rapidly in response to either a single 2 Gy dose fraction or a 10 Gy dose of γ-radiation in HCT-116 cells. They found that miR-451a inhibits tumor cell proliferation and attenuated surviving fraction in longer-term cultures. Conversely, inhibition of miR-451a increased proliferation, tumorsphere formation, and surviving fraction of tumor cells. Using a bioinformatics approach, we identified four genes, CAB39, EMSY, MEX3C, and EREG as targets of miR-451a. Transfection of miR-451a decreased both mRNA and protein levels of these targets (Ruhl et al., 2018).

Ubiquitin-conjugating enzyme E2C (UBE2C) has been implicated as a key regulator of cell cycle progression in various malignancies (Liu et al., 2019). Zhang et al. (2018) found that UBE2C was aberrantly upregulated in rectal carcinoma, whereas siRNA-mediated knockdown of UBE2C significantly inhibited cell viability, proliferation, and induced apoptosis *in vitro*. In xenograft mice, tumor growth was markedly suppressed upon UBE2C silencing. Forced expression of miR-381 in HR-8348 cells dramatically inhibited UBE2C expression at the protein level, which clearly revealed that miR-381 post-transcriptionally modulated UBE2C in rectal carcinoma. Thereby, the aberrant proliferation and apoptosis can be attributed to the high expression of UBE2C, which can be ameliorates by miR-381 expression (Zhang et al., 2018). Oner et al. (2018) used mice model with disruption of miR-34a and/or TP53 specifically in intestinal epithelial cells, which is characterized with larger and more colorectal tumors, increased invasion of surrounding tissue and metastasis to lymph nodes, than control mice. Cells in tumors from these mice had decreased apoptosis and increased proliferation compared to tumor cells from control mice. For mechanisms, they found that CD3bT cells, bacterial infiltration, and expression of Mucin1 were increased in tumors of mice model. Administration of tiplaxtinin or anti-IL6R antibody to these mice model inhibits proliferation of cancer cells, and reduced colorectal tumor invasion and metastasis (Oner et al., 2018).

### Progression

Tumor progression, the final phase of carcinogenesis, is characterized by increased invasiveness and growth (Jiang et al., 2018; Shi et al., 2018). Downregulation of miR-148a and miR-625-3p is associated with tumor budding in tumor specimens from 54 patients with a first-time diagnosed colorectal cancer, determined by real-time quantitative (qRT)-PCR (Baltruskeviciene et al., 2017).

Huang et al. (2015) found that miR-19a was upregulated in colorectal cancer surgical tissues and high expression of miR-19a was significantly associated with lymph node metastasis. They further analyzed miR-19a lymph node metastasis signature in surgical tissue specimens from an external validation cohort of 311 colorectal cancer cases of The Cancer Genome Atlas (TCGA); and discovered that miR-19a was significantly associated with lymph node metastasis in rectal cancer. *In vitro*, overexpression of miR-19a in human HCT 116 and Caco-2 cells promoted cell invasion and EMT. miR-19a overexpression is correlated with lymph node metastasis and participates in tumor necrosis factor (TNF)-α-induced EMT in colorectal cancer (Huang et al., 2015). Bioinformatic analysis identified that miR-4728-3p as a regulator of 3 proteins (CAV1, THBS2, and COL1A2) is involved in focal adhesion signaling, evidenced from samples of patients. Quantitative realtime polymerase chain reaction (qRT-PCR) of tissue specimens validated that miR-4728-3p is likely a significant tumor suppressor in ulcerative colitis-associated colon carcinogenesis via regulating adhesion signaling (Pekow et al., 2017). The miR-195 mimic was transfected into 2 types of human rectal cancer cells (SW837 and SW1463). The results revealed that insulin-like growth factor 1 (IGF1) was predicted as a potential target of miR-195 by Targetscan7.2, and the result was verified by dual-luciferase reporter assay. The phosphorylation of PI3K and Akt was significantly inhibited in the mimic group. The tumor suppressive ability of miR-195 in rectal cancer cell proliferation and metastasis was mediated by blocking IGF1 expression and inhibiting the PI3K/Akt pathway (Wang et al., 2019).

The main limitation from these studies is the *in vitro* model used, or clinical samples from patients, which was analyzed by qRT-PCR. *In vivo* or human model are lacked, which fails to provide a comprehensive understanding for readers. Further *in vivo* animal and clinical studies are needed to elucidate this problem.

### Other Mechanisms

Studies have suggested that other mechanisms are also involved in colorectal cancer. Hua et al. (2017) used TCGA database to explore mRNA expression profiling and corresponding DNA methylation data. Eventually, 50 differentially expressed (DE) mRNAs DEmRNAs (39 down- and 11 upregulated DEmRNAs) with hypermethylation, synchronously negatively targeted by DEmiR, were identified through the correlation analysis among 446 genes with aberrant methylation and 668 miR, evidenced from rectal adenocarcinoma tumor samples. They further used qRT-PCR and microarray analyses to validate and identified 7 genes of SORCS1, PDZRN4, LONRF2, CNGA3, HAND2, RSPO2, and GNAO1 with hypermethylation and negatively regulation by DEmiR, the seven of which might contribute to the tumorigenesis of rectal adenocarcinoma (Hua et al., 2017). Stable expression of SNAI1 in DLD1 and HCT116 cells downregulates miR-145 expression compared to control cells. Using a miR-145 luciferase reporter assay, Zhu et al. (2018) determined that ectopic SNAI1 significantly repressed the miR-145 promoter in DLD1-SNAI1 and HCT116-SNAI1 cells, accompanied by reduced radiotherapy sensitivity and, conversely, miR-145 replacement significantly enhanced radiotherapy sensitivity. Furthermore, miR-145 replacement decreased cancer stem cells-related transcription factor expression, spheroid formation, and radiation resistance (Zhu et al., 2018).

Epithelial-mesenchymal transition is correlated with poor outcomes in various cancers. Compared to parental DLD1 colon cancer cells, 5FU-resistant (5FUr) DLD1 cells demonstrated features of EMT, including enhanced invasion and migration, suppressed E-Cadherin expression, and 2-fold increased SNAI2 expression. DLD1 and HCT116 cells with stable expression of SNAI2 (DLD1/SNAI2; HCT116/SNAI2) also demonstrated EMT features such as the decreased E-Cadherin, as well as significantly decreased miR-145 expression, as compared to control empty vector cells. Based on a miR-145 luciferase promoter assay, we demonstrated that SNAI2 repressed activity of the miR-145 promoter in the DLD1 and HCT116 cells, accompanied by an enhanced 5FU sensitivity (Findlay et al., 2014). miR-9 has a tumor-suppressive role in colorectal cancer. miR-9 inhibits the IGF1 receptor (IGF1R)/Src signaling and cyclin B1 and N-cadherin, whereas upregulates E-cadherin signaling.

## Theranostic Value of miR in Rectal Cancer

There still remain urgent issues and questions to the theranostics of colorectal cancer. Firstly, there is lack of effective application of non-invasive biomarkers for early diagnosis. Fecal occult blood test and colonoscopy are currently common method of early diagnosis, which may reduce the incidence and mortality of colorectal cancer in a population more than 50 years old (Mandel et al., 2000). Unfortunately, fecal occult blood test has a low sensitivity and specificity. Colonoscopy is the gold standard for early diagnosis of colorectal cancer whereas it is difficult in practice, costly, and at risk for complications. Secondly, there is still a lack of indexes for effective prognosis and chemoradiotherapy response. At present, the clinical basis is mainly based on clinicopathological indicators (such as tumor size and lymph node) and TNM stage for guidance, which cannot dynamically monitor disease outcome and predict tumor response and the prognosis of patients of a large heterogeneity (Burke, 2004). Thus novel theranostic molecular signalings are needed to elucidate the problems.

### Diagnosis

Eriksen et al. (2016) performed high-throughput miR profiling on 10 pairs of laser micro-dissected rectal cancer tissue and adjacent stroma from rectal cancer patients. A global mean expression normalization strategy was applied to determine the most stably expressed miR for subsequent validation. From the miR profiling experiment, miR-645, miR-193a-5p, miR-27a, and let-7g were identified as stably expressed, both in malignant and stromal tissue, thereby they recommend the mean expression of miR-27a, miR-193a-5p, and let-7g as normalization factor, when performing miR expression analyses by RT-qPCR on rectal cancer tissue (Eriksen et al., 2016). Expression of genes and miR from tumor and non-tumor samples obtained from surgical treatment of 50 colorectal cancer patients were tested by qRT-PCR. They concluded that an increased expression of miR -21 and 203 in tumor samples in relation to non-tumor samples was found. The expression of miR-203 was progressively lower in relation to the TNM staging and was higher in the patient group in clinical remission (Carvalho et al., 2017). To determine the mechanisms of the malignant transformation, Bao et al. (2014) conducted miR array on the colonic epithelial cells from the 3-month Muc22/2 and ^+/+^ mice. Based on relevance to cytokine and cancer, 4 miR (miR-138, miR-145, miR-146a, and miR-150) were validated and were found significantly downregulated in human colitis and colorectal cancer tissues. The network of the targets of these miR was characterized, and interestedly, miR-associated cytokines were significantly increased in Muc22/2mice (Bao et al., 2014).

The expression of some miRs (e.g., miR-145, miR-150, and miR-146a) is also changed in other diseases. Wei et al. (2017) used qRT-PCR to compare plasma miR-145 levels in 120 patients with cervical cancer, and 120 healthy volunteers. The results demonstrated that low levels were significantly associated with poor cancer differentiation, lymph node metastasis, and human papillomavirus (HPV). Cervical cancer patients who achieved complete response to radiotherapy had higher plasma miR-145 levels than incomplete responders. Receiver operating characteristic curve (ROC) analysis confirmed that plasma miR-145 is a candidate biomarker for detecting cervical cancer and differentiating complete responders from incomplete responders (Wei et al., 2017). Yan et al. (2017) carried out a meta-analysis that included 7 original studies (data from PubMed, Embase, and Web of Science), and discovered that miR-150 may be a potential non-invasive tumor marker for various human cancers (leukemia, colorectal, hepatocellular, and lung cancer) (Yan et al., 2017). Besides, a total of 73 patients with papillary thyroid carcinoma were enrolled. Carcinoma samples were obtained from each patient, and adjacent tissues were used as control samples to determine expression levels of miR-146a and miR146b by semi-quantitative RT-PCR. The results showed that the expression levels of miR-146a and miR-146b in carcinoma tissues were significantly higher than the levels in cancer-free tissues. The relative expression levels of miR-146a and miR-146b in cancerous tissues might be associated with the pathological type and presence or absence of lymph node metastasis (Qiu et al., 2017).

### Staging

Wang et al. (2009) applied qRT-PCR and found that miR-31 expression was positively related to advanced TNM stage and deeper invasion of tumors in 98 primary colorectal cancer specimens. miR-145 was downregulated in both colon and rectal cancer. miR-143 was only downregulated in tumor specimens from colon cancer but not in rectal cancer, which suggests that the miR-143 and miR-145 may play a certain role in the development of colon and/or rectal cancers (Wang et al., 2009). Slattery et al. (2015) used the Agilent Human miR Microarray V19.0 to generate miR data following a stringent quality control protocol. They discovered that five miR are correlated with more advanced stage from tumor specimens of colorectal cancer. hsa-miR-145-5p and hsa-miR-31-5p are associated with increased expression with more advanced tumor stage; hsa-miR-200b-3p, hsa-miR-215, and hsa-miR-451a have decreased expression with more advanced tumors (Slattery et al., 2015).

The expression of some miRs (e.g., hsa-miR-145-5p and miR-155) is also changed in other diseases. Inamoto et al. (2018) used 3D-Gene miR labeling kit to determine miR expression in patients’ tumor samples with an aggressive phenotype or non-aggressive phenotype. They discovered that deregulation of hsa-miR-145-5p was determined in urothelial carcinoma of the bladder (UCB) patients with aggressive phenotype compared with non-aggressive subject. The survival status and tumor miR expression of all 84 UCB patients were ranked according to the prognostic score values. Of nine miR, hsa-miR-145-5p were shown to be protective (Inamoto et al., 2018). miR-155 is a diagnostic and prognostic marker in both hematological and solid malignancies, including breast cancer, colorectal carcinoma, pancreatic carcinoma. miR-155 is considered as a typical multifunctional miR including its role as oncomiR (cancer-associated miR). Expression of miR-155 is upregulated in cells with high proliferative activity and decreased apoptotic capability (Jurkovicova et al., 2014).

### Therapy

There is a significant upregulation of miR-143 and miR-145 in post-therapeutic tumor tissue compared to pre-therapeutic tumor tissue. Patients with a low intratumoral post-therapeutic expression significantly have a worse response to neoadjuvant therapy compared to rectal cancer patients with a high expression of miR-145, determined by macrodissected tumor tissue (Drebber et al., 2011). Neoadjuvant chemoradiotherapy (nCRT) following surgery significantly improves the survival rate of patients with rectal cancer, whereas it has significant adverse symptoms and high medical costs. Du et al. (2018) used GEO database and acquired data from a complete response (CR) and incomplete response (IR) group. The results demonstrated that a total of 36 upregulated and 5 downregulated miR were identified from fresh frozen biopsies between the two groups. Among these differentially expressed miR, miR-548c-5p, miR-548d-5p, and miR-663a were significantly associated with a CR to nCRT (Du et al., 2018). Besides, RNA from pretreatment endoscopy biopsies from 96 rectal cancer patients treated with preoperative chemoradiotherapy were studied. An 8-miR chemoradiotherapy-response signature is identified: miR-21, let-7b, miR-99b, let-7e,miR-183, miR-328, miR-375, and miR-483-5p. In the validation phase, miR-99b, miR-21, and miR-375 act as chemoradiotherapy response-related miR whereas miR-328 and let-7e emerge as prognostic markers for disease-free survival and overall survival. ROC curve analysis revealed that the combination of miR-21, miR-99b, and miR-375 had the best capacity to distinguish patients with maximum response (TRG4) from others (Campayo et al., 2018).

The expression of these miRs (e.g., miR-145, miR-148, miR-375, etc.) is also changed in other diseases, mainly in breast cancer. Gonzalez-Villasana et al. (2019) isolated exosomes from serum of breast cancer patients and healthy donors, and observed that a round shape of exosomes with a mean size of 119.84 nm in breast cancer patients and 115.4 nm in healthy donors. They detected miR-145, miR-155, and miR-382 in the exosomes isolated from serum of breast cancer patients and healthy donors. The results show that exosomes isolated from the serum of breast cancer patients and healthy donors contains miR-145, miR-155, and miR-382 but not in a selective manner in breast cancer patients (Gonzalez-Villasana et al., 2019). Spindlin 1 (SPIN1) was significantly elevated in drug-resistant breast cancer cell lines and tissues, compared with the chemosensitive ones. SPIN1 enhanced adriamycin resistance of breast cancer cells *in vitro*, and downregulation of SPIN1 by miR could decrease adriamycin resistance *in vivo*. Notably, SPIN1 was identified as a direct target of the miR-148/152 family (miR-148a-3p, miR-148b-3p, and miR-152-3p). As expected, miR-148a-3p, miR-148b-3p, or miR-152-3p could increase adriamycin sensitivity in breast cancer cells (MCF-7, MDA-MB-231, and MDA-MB-468) *in vitro*.

### Prognosis

High levels of miR-31 are reported overexpressed in 34.2% of rectal cancer. Its overexpression predicts poor overall survival and pathological response, evidenced from biopsies from 78 patients diagnosed with locally advanced rectal cancer (Carames et al., 2016). Expressions of 2,555 miR were examined in 20 pairs of rectal tumors specimens and matched non-malignant tissues by 3D-Gene Toray microarray. Kral et al. (2018) identified rectal cancer-specific miR signature that distinguishes responders from non-responders to adjuvant chemotherapy. A predominant part of identified miR was represented by the members of miR-17/92 cluster. Upregulation of miR-17, -18a, -18b, -19a, -19b, -20a, -20b, and -106a in tumor was associated with higher risk of tumor relapse and their overexpression in rectal cancer cell lines stimulated cellular proliferation (Kral et al., 2018). Moreover, miR-21 (Kang et al., 2015; Mima et al., 2016), let-7g (Salendo et al., 2013), let-7a (Dou et al., 2016), miR-31 (Li et al., 2018) are shown to be involvement in the prognosis of colorectal cancer (Table 1).

**TABLE 1:** Mechanisms of miR in colorectal cancer.

Types	Model/Methods	Mechanisms	Key miR	Evidence	References
Colorectal cancer	Bioinformatics via open database	Carcinogenesis	hsa-miR-630, hsa-miR-100 and hsa-miR-99a	hsa-miR-630, hsa-miR-100 and hsa-miR-99a are involved in colorectal carcinogenesis and needed experimental validation.	Pradhan et al., 2015
Colorectal cancer	Tumor mucosa	Carcinogenesis	miR-155, miR-34a, and miR-200c	miR-34a and miR-200c were significantly upregulated in rectum compared to normal mucosa. In colon, the higher expression of three miRNAs was seen, however, without significant difference.	Wang et al., 2012
Rectal cancer	HRC-9698 cell	Proliferation and apoptosis	Antisense miRNA	Antisense microRNA targeting survivin can inhibit HRC-9698 cell proliferation and induce its apoptosis.	Dai et al., 2015
Rectal cancer	SW837 and SW1463 cell	Proliferation and progress	miR-144	The supplementation of ROCK1 markedly restored the cell migration and proliferation, the process of which can be inhibited by miR-144.	Cai et al., 2015
Rectal cancer	HCT-116 cell	Proliferation	miR-451a	miR-451a inhibits tumor cell proliferation and attenuated surviving fraction of HCT-116 cells.	Ruhl et al., 2018
Rectal carcinoma	Xenograft B-NSG nude mice	Proliferation	miR-381	Forced expression of miR-381 in HR-8348 cells dramatically inhibited UBE2C expression and tumor growth.	Zhang et al., 2018
Colorectal cancer	VillinCre mice with disruption of miR-34a and/or TP53 specifically in intestinal epithelial cells	Proliferation and apoptosis	miR-34a	Cells in tumors from these mice had decreased apoptosis and increased proliferation compared to tumor cells from control mice.	Oner et al., 2018
Colorectal cancer	Tumor specimens from 54 patients	Progression	miR-148a and miR-625-3p	Downregulation of miR-148a and miR-625-3p is associated with tumor budding in tumor specimens from 54 patients with a first-time diagnosed colorectal cancer, determined by real-time quantitative qRT-PCR.	Baltruskeviciene et al., 2017
Colorectal cancer	Surgical specimens from patients	Progression	miR-19a	miR-19a was significantly associated with lymph node metastasis in rectal cancer. *In vitro*, overexpression of miR-19a in human colorectal cell lines promoted cell invasion and EMT.	Cai et al., 2015
Colonic neoplasia	Tumor specimens from patients	Progression	miR-4728-3p	miR-4728-3p is likely a significant tumor suppressor in ulcerative colitis-associated colon carcinogenesis via regulating adhesion signaling.	Pekow et al., 2017
Rectal cancer	SW837 and SW1463	Proliferation and progression	miR-195	The tumor suppressive ability of miR-195 in rectal cancer cell proliferation and metastasis was mediated by blocking IGF1 expression and inhibiting the PI3K/Akt pathway.	Wang et al., 2019
Rectal cancer	DLD1 and HCT116 cell	Radiotherapy sensitivity	miR-145	miR-145 replacement decreased cancer stem cells-related transcription factor expression, spheroid formation, and radiation resistance.	Zhu et al., 2018
Rectal cancer	DLD1 and HCT116 cell	Chemotherapy sensitivity	miR-145	SNAI2 repressed activity of the miR-145 promoter in the DLD1 and HCT116 cells, accompanied by an enhanced 5FU sensitivity.	Findlay et al., 2014

The expression of these miRs above is also changed in other diseases. Circ-ITCH is a circRNA generated from several exons of itchy E3 ubiquitin protein ligase (ITCH) and tumor suppressor. Circ-ITCH, is down-regulated in bladder cancer tissues and cell lines. Bladder cancer patients with low circ-ITCH expression had shortened survival. Enforced- expression of circ-ITCH inhibited cells proliferation, migration, invasion and metastasis both *in vitro* and *in vivo*. Mechanistically, we demonstrated that circ-ITCH up-regulates the expression of miR-17 and miR-224 target gene p21 and PTEN through ‘sponging’ miR-17 and miR-224, which inhibited the aggressive biological behaviors of bladder cancer (Yang et al., 2018). The expression of miR-31 in human laryngeal cancer TU686 cells, human nasopharyngeal carcinoma CNE-2 cells, and normal human oral keratinocyte (NHOK) epithelial cells was detected via qRT-PCR. Results reveal that the expressions of miR-31 in TU686 and CNE-2 cell lines were significantly higher than that in normal human oral keratinocyte (NHOK) epithelial cells. Compared with those in the negative control group, the proliferation and invasion abilities of cells transfected with miR-31 mimics were notably enhanced, and those of cells transfected with anti-miR-31 were significantly reduced. In addition, miR-31 mimics significantly reduced ARID1A expression and anti-miR-31 increased its expression. The expression of miR-31 in tumor tissues of HNSCC patients was remarkably higher than that in tumor-adjacent normal tissues (Table 2; Qiang et al., 2019).

**TABLE 2:** Theranostic value of miR in rectal cancer.

Types	Model/methods	Theranostic value	Key miR	Evidence	References
Rectal cancer	Tumor specimen from patients	Diagnosis	miR-645, miR-193a-5p, miR-27a and let-7g	miR-645, miR-193a-5p, miR-27a and let-7g were identified as stably expressed, both in malignant and stromal tissue. The mean expression of miR-27a, miR-193a-5p and let-7g as normalization factor.	Eriksen et al., 2016
Colorectal cancer	Tumor specimen from patients	Diagnosis	miR -21 and 203	An increased expression of miR -21 and 203 in tumor samples in relation to non-tumor samples was found. The expression of miR-203 was progressively lower in relation to the TNM staging and was higher in the patient group in clinical remission.	Carvalho et al., 2017
Colorectal cancer	Muc22/2 and ^+/+^ mice	Diagnosis	miR-138, miR-145, miR-146a, and miR-150	miR-138, miR-145, miR-146a, and miR-150 were validated and were found significantly downregulated in human colitis and colorectal cancer tissues.	Bao et al., 2014
Colorectal cancer	Tumor specimen from patients	Staging	miR-143 and miR-145	miR-145 was downregulated in both colon and rectal cancer. miR-143 was only downregulated in tumor specimens from colon cancer but not in rectal cancer.	Wang et al., 2009
Colorectal cancer	Public database	Staging	hsa-miR-145-5p, hsa-miR-31-5p, miR-200b-3p, hsa-miR-215, and hsa-miR-451a	hsa-miR-145-5p and hsa-miR-31-5p is associated with increased expression with more advanced tumor stage; hsa-miR-200b-3p, hsa-miR-215, and hsa-miR-451a have decreased expression with more advanced tumors.	Slattery et al., 2015
Rectal cancer	Tumor specimen from patients	Therapy	miR-145	Patients with a low intratumoral post-therapeutic expression significantly have a worse response to neoadjuvant therapy compared to rectal cancer patients with a high expression of miR-145, determined by macrodissected tumor tissue.	Drebber et al., 2011
Rectal cancer	GEO database	Therapy	miR-548c-5p, miR-548d-5p, and miR663a	miR-548c-5p, miR-548d-5p, and miR-663a were significantly associated with a CR to nCRT.	Du et al., 2018
Rectal cancer	Tumor specimen from patients	Therapy	miR-99b, miR-21, miR-328, and miR-375	miR-99b, miR-21,and miR-375 act as chemoradiotherapy response-related miR whereas miR-328 and let-7e emerge as prognostic markers for disease-free survival and overall survival.	Campayo et al., 2018
Rectal cancer	Tumor specimen from patients	Prognosis	miR-31	High levels of miR-31 are reported overexpressed in 34.2% of rectal cancer. Its overexpression predicts poor overall survival and pathological response.	Carames et al., 2016
Rectal cancer	Tumor specimen from patients	Prognosis	miR-17, -18a, -18b, -19a, -19b, -20a, -20b and -106a	Upregulation of miR-17, -18a, -18b, -19a, -19b, -20a, -20b, and -106a in tumor was associated with higher risk of tumor relapse and their overexpression in rectal cancer cell lines stimulated cellular proliferation.	Kral et al., 2018

## Further Perspectives

### Circulating miR in Rectal Cancer

Circulating miR stably exists in the serum, plasm, and other body fluids. Acting as a type of non-invasive biomarker, circulating miR can be used to provide guidance for the early diagnosis, monitoring the curative effects, and prognosis estimation. Lawrie et al. (2008) discovered that miR-21 and miR-155 are remarkably increased in serum of diffuse large B-cell lymphoma patients in 2008, suggesting the potential theranostic values of circulating miR in cancer. Research shown that circulating miR are mostly encapsulated in microvesicles or apoptosis bodies (Zernecke et al., 2009; Zhang et al., 2010), which can protect miR from degradation. Secondly, the structure of miR in the blood may be modified by such modifications as methylation and adenylation to make it resistant to nuclease degradation. Furthermore, miR may be protected by binding to certain protein molecules or other lipids.

Circulating miR are promising candidates, whereas circulating miR analyses in rectal cancer are rare. miR-17, miR-20a, miR-18b, miR-193a-3p, and miR-31, are significantly reduced in pretreatment plasma of rectal cancer patients. Extracellular vesicles from colorectal cancer are exosomes containing the oxygen-sensitive miR 486-5p, 181a-5p and 30d-5p, which are retrieved as circulating markers of high-risk locally advanced rectal cancer (Bjornetro et al., 2019). miR-155, miR-34a and miR-29a are downregulated in patients determined with colorectal by qRT-PCR assay. In plasma of patients with rectal cancer, miR-221 expression is higher than the controls. The reduced expression of miR-18b and miR-20a during CRT was found to be significantly associated with postoperative lymph node negativity. Furthermore, advanced stage is also linked to higher miR-221 expression compared to early stage (Azizian et al., 2015).

### Life Style and Colorectal Cancer

Life style or dietary habit is shown to act in the development of colorectal cancer. Levels of oncogenic mature miR, including miR-21 and miR17-92 miR cluster, increase in the rectal mucosa with the high red meat diet (HRM), whereas the HRM + butyrylated resistant starch diet restores miR17-92 miR to baseline levels (Humphreys et al., 2014). Malcomson et al. (2017) screened 1008 miR in pooled post-intervention rectal mucosal samples from participants allocated to the double placebo group and those supplemented with both resistant starch and polydextrose. Resistant starch can increase the risk for colorectal cancer by enhancing the expression of miR-32. miR-32 expression increases in the rectal mucosa of participants supplemented with resistant starch + polydextrose compared with placebo. miR-32 is involved in the regulation of processes such as cell proliferation that are dysregulated in colorectal cancer (Malcomson et al., 2017). Mullany et al. (2017) studied 1,447 colorectal cancer cases with normal mucosa and carcinoma miR expression data along with alcohol consumption data. They analyzed long-term and long-term and current (LTC) alcohol use for beer, liquor, and wine with miR expression between paired carcinoma and normal colon and rectal tissues. They discovered that expression of 84 miR is associated significantly with long-term and current wide use in normal rectal mucosa. Higher expression of hsa-miR-210 and hsa-miR-92a-1-5p significantly worsens all-cause mortality. These miR are downregulated across levels of long-term and current wine consumption (Mullany et al., 2017).

### Other Perspectives

For clinical studies, Humphreys et al. (2014) examined whether a HRM diet altered miRNA expression in rectal mucosa tissue of healthy volunteers, and if supplementation with butyrylated resistant starch (HRM + HAMSB) modified this response. Fecal butyrate increased with the HRM + HAMSB diet. Levels of oncogenic mature miRNAs, including miR17-92 cluster miRNAs and miR-21, increased in the rectal mucosa with the HRM diet, whereas the HRM + HAMSB diet restored miR17-92 miRNAs, but not miR-21, to baseline levels. Elevated miR17-92 and miR-21 in the HRM diet corresponded with increased cell proliferation, and a decrease in miR17-92 target gene transcript levels, including CDKN1A (Humphreys et al., 2014). Diet-induced obesity increased (and calorie restriction decreased) the number of colon tumors and proliferation. Diet-induced obesity decreased (and calorie restriction increased) apoptosis. miR including miR-425, miR-196, miR-155, miR-150, miR-351, miR-16, let-7, miR-34, and miR-138 were differentially expressed between the dietary groups (Olivo-Marston et al., 2014).

## Conclusion

Despite major advances in clinical treatment, mortality from colorectal cancer remains high and 40–50% of patients eventually die as a result of their disease (Kuipers et al., 2015). The future of cancer surgery for colorectal disease aims to minimize surgical trauma and preserving organ function (Kuipers et al., 2015). There are obvious limitations and advantages to target miR or use miRs as diagnostic marker or use miRs to monitor therapeutic response. miR bears good sensitivity and specificity compared to common clinical diagnosis. And some clinical studies and cohort studies have demonstrated the therapeutic and diagnosis values of miR. However, instability does exist when extract miR (Bravo et al., 2007; Bernardo et al., 2012). Besides, there are a plenty of downstream signaling targets of miR, thereby distinguishing the specific targets of a certain miR is a huge workload (Khatri et al., 2012).

Recent studies have suggested an elaborate network involving miR that confer potential directions for inherent mechanisms of colorectal cancer. However, the internal mechanisms remains unclear, and novel avenues may include: (i) exploring upstream/downstream signaling pathways to provide a comprehensive picture of the miR network; (ii) exploring the exogenous drugs, activators/inhibitors to regulate the activity of miR (iii) clinical trials or cohort studies of miR-targeted drugs are needed to verify their efficacy in neoplasms.

## Disclosure

All authors declare no competing interests, including but not limited to: (1) all authors have no financial or other interest in the product or distributor of the product. (2) There is no relation between any author and the manufacturer or distributor of the product. (3) There are no other kinds of associations, such as consultancies, stock ownership, or other equity interests or patent-licensing arrangements, also must be disclosed. (4) This study has no relationship with any pharmaceutical factories or commercial groups. (5) No medical writers or editors were involved in this article.

## Author Contributions

TL and XW: conceptualization, supervision, and project administration. FY: validation and funding acquisition. XW, FY, and LW: investigation. GS and JL: data curation. XW: writing –original draft. TL, FY, and YW: writing – review and editing. MQ: visualization. All authors contributed to the article and approved the submitted version.

## Conflict of Interest

The authors declare that the research was conducted in the absence of any commercial or financial relationships that could be construed as a potential conflict of interest.
